# Bibliometric research analysis of molecularly imprinted polymers (MIPs): evidence and research activity dynamics

**DOI:** 10.1007/s11356-023-30752-w

**Published:** 2023-11-07

**Authors:** Nikolaos Mittas, Despina A. Gkika, Konstantinos Georgiou, Abdullah N. Alodhayb, Naglaa AbdelAll, Ghada A. Khouqeer, George Z. Kyzas

**Affiliations:** 1 Hephaestus Laboratory, Department of Chemistry, International Hellenic University, 65404 Kavala, Greece; 2 School of Informatics, Aristotle University of Thessaloniki, 54124 Thessaloniki, Greece; 3 Department of Physics and Astronomy, College of Science, King Saud University, 11451 Riyadh, Saudi Arabia; 4 Department of Physics, College of Science, Imam Mohammad Ibn Saud Islamic University (IMSIU), Riyadh, Saudi Arabia

**Keywords:** Bibliometric analysis, Scientometrics, Molecularly imprinted polymers, Molecular imprinting

## Abstract

The escalating issue of water pollution has become a worldwide issue that has captured the attention of numerous scientists. Molecularly imprinted polymers (MIPs) have emerged as adaptable materials with exceptional attributes, including easy synthesis, low cost, remarkable durability, long life, and accessibility. These attributes have motivated researchers to develop novel materials based on MIPs to tackle hazardous contaminants in environmental matrices. The purpose of this paper was to conduct a bibliometric analysis on MIPs’ publications, in order to shed light on the developments and focus points of the field. The selected publications were obtained from Scopus database and subjected to a filtering process, resulting in 11,131 relevant publications. The analysis revealed that the leading publication source (journal) is *Biosensors and Bioelectronics*; the mostly employed keywords are solid-phase extraction, electrochemical sensor, and molecular recognition; and the top contributing countries are China, Iran, and the USA. The Latent Dirichlet Allocation (LDA) algorithm was used for extracting thematic axes from the textual content of the publications. The results of the LDA model showcase that the topic of synthesis and performance of MIPs for environmental applications can be considered as the most dominant topic with a share value of 72.71%. From the analysis, it can be concluded that MIPs are a cross-disciplinary research field.

## Introduction

Starting from the early 1970s, there has been a focus on enhancing the quality of effluent water, particularly in industrialized countries, through public wastewater treatment sites (Dhaka et al. 2019). Environmental pollution poses a very significant and daunting challenge to humanity (Nasrollahi et al. 2020) and involves the existence of numerous pollutants, such as dyes, heavy metals, organic molecules, and more, all of which have the potential to impact the environment (Ma et al. 2019). The continuous release of chemical substances through various activities has negatively affected both the environment and human health. As a result, it has become imperative to develop simple, quick, cost-effective, and efficient methods for the isolation or extraction of such substances from soil or water (Azizi and Bottaro 2020). So far, numerous works have explored adsorption and photocatalytic processes. Sample treatment is one of the most crucial phases of chemical analysis, mainly concentrating on the extraction of interfering compounds found in the sample and the preconcentration of analytes to reach the appropriate sensitivity levels (Torres-Cartas et al. 2020). Molecular imprinting is one of the various methods to accomplish this. The significance of molecular imprinting lies in the need to be able to selectively bind specified target pollutants. The capacity to extract and reuse substances (molecules, ions etc.) in either their original or somewhat diversified form in any kind of environmental application has great value. This ability, described as molecular recognition, entails the selective identification of a pre-specified material that is mixed with multiple other similar materials and is fundamental to biochemical processes, taking place everywhere in nature (Kyzas and Bikiaris 2015).

*Molecularly imprinted polymers* (MIPs), in particular, have been in the spotlight as emerging materials for pollutant elimination. This is due to their predictable structure, exceptional recognition ability, and adaptability in tackling environmental pollutants (Bagheri et al. 2021). Moreover, MIPs have been increasingly used in recent years in an effort to achieve better selectivity (Chen et al. 2016), using interactions that resemble those of antigens and antibodies in living organisms. MIPs comprise of densely cross-linked polymeric porous materials that possess attributes similar to those of the targeted material. The approach involves the building of sites within a synthetic polymer that can recognize the target counterparts (Fizir et al. 2020).

The interest of the research community on the field of MIPs is not a novel concept, as it dates back to the 1930s (Sajini and Mathew 2021). From then on, MIP elements have conceptually attracted attention (Strikovsky et al. 2000) due to their extensive use, as an amalgamation of desirable attributes, such as high physical stability, robustness, excellent reusability, and low-cost synthesis (Metwally et al. 2021). This has led, in turn, to an exponential growth in the publication rate regarding MIPs technology. Many scholars that have previously conducted similar research utilized a theoretical review approach. Nicholls et al. (Nicholls et al. 2022) assessed prior works regarding the role of molecular dynamics in the advancement of molecular imprinting, while Anja Mueler (Mueller 2021) studied how the cross-linking density controls the physical properties and the selectivity of the produced MIPs. Garnier et al. (Garnier et al. 2021) illustrate how hybrid MIPs can be applied in enhanced imaging and treatments, promoting patient care.

Despite theoretical reviews’ usefulness in comprehending certain points of view of a research subject, bibliometric methods provide a different perspective when the domain to be covered is as vast as that of MIPs (Grant and Booth 2009). In such cases, bibliometric analyses are more appropriate because they can offer quantitative indicators about the contents, structure, and trends of the studied field (Li et al. 2021). Until now, to the best of our knowledge, no documented bibliometric study exists that specifically addresses the publication research activity related to MIPs. The present study aims at identifying the most prominent trends and to uncover potential knowledge gaps or research priorities where further research is required.

The novelty of this work is threefold: Firstly, in order to deal with the inter-disciplinary nature of the research and provide a macroscopic overview on its main characteristics, we designed a research framework that can adjust complexity of the issue combining drivers and approaches, to fulfill the objectives of the bibliometric analysis. Secondly, its mission is to explore the current literature and provide up-to-date (and easy to compare) information on MIP work. Thirdly, it aims to uncover relationships in the study field through statistical methods.

Therefore, the main objective is to introduce a research framework and provide a perspective of the concept of MIP research, by exploring the current status of comprehending the research activity of MIPs referred to in published scientific works. In light of the above, this work proceeded to (a) conduct a MIP scientific literature performance analysis and (b) apply a science mapping on MIPs within the 1990–2021 period. The topic of MIPs is growing year by year, and the bibliometric research analysis for these chemical materials will drastically help to understand the “future” and trend of these materials.

The structure of this article is organized as follows: the second section presents the methodological workflow used throughout this study. The third section presents the results extracted from the bibliometric analysis conducted on the collection of documents, offering answers to the posed research questions and presents the threats to validity. The fourth section discusses the related findings and concludes this work, summarizing its innovation, contribution, and potential future directions.

## Methodology

**Fig. 1:**
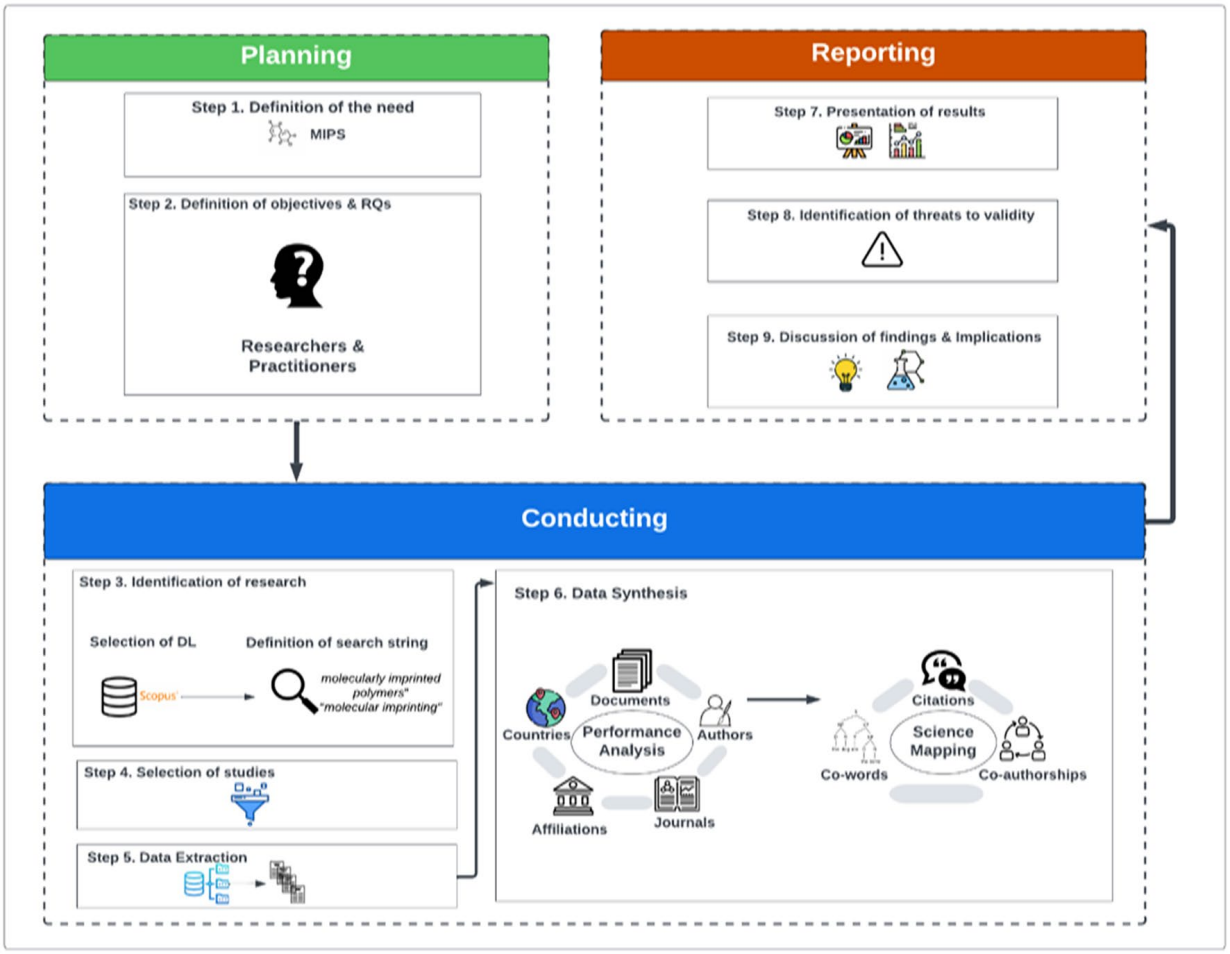
Methodology and steps followed in the study

In this section, we present in detail the methodology followed in order to meet the objectives of the current study. We decided to design a protocol with predefined, phases, steps, and activities adapting the general principles of the process followed during the conduction of other well-known types of secondary studies that are the *systematic literature reviews* (SLRs) (Kitchenham and Charters n.d.) and *systematic mapping studies* (SMS) (Petersen et al. 2015). The protocol comprises three key phases that are the (*i*) *planning*, (*ii*) *conducting*, and (*iii*) *reporting* phases (Fig. 1). Each one of the above phases involves a set of steps with related activities, tasks, and decisions that should be defined in advance to fulfill the objectives of a bibliometric analysis study.

### Planning phase

The *planning phase* is dedicated to two specific steps involving (*i*) the definition of the necessity and motivation for conducting a bibliometric analysis study on the examined topic (step 1) and (*ii*) the formation of the research objectives and their associated research questions fulfilling the goals of this study (step 2).

#### Define the need for the bibliometric study (step 1)

The notion of MIPs is characterized by complexity. Firstly, MIP process is an intricate issue, based on its content. Considerable efforts have been dedicated to test models that aim at framing the concept and defining its perspectives. However, a limited consensus has been reached on the cause and the consequences of MIP technology. Secondly, an additional source of complexity lies in the fact that MIP technology is a multi-dimensional research topic. As previously mentioned, the concept was first used to recognize chemical and biological molecules. However, the concept of MIPs has not only been explored for the recognition of molecules but has also been adopted in separation science, drug delivery purification, artificial antibodies, chemo-biosensing, enantiomeric recognition, catalysis, and degradation (Arabi et al. 2021; Chen et al. 2016). Thirdly, the intricacy of MIPs is reinforced by a wide variety of academic fields interested in the relevant research, such as polymer and material chemistry, biochemistry, economics, and other multidisciplinary approaches (Liu et al. 2019), promoting diverse exploration and evaluation approaches.

These cross-disciplinary insights have resulted in very different approaches for exploring and assessing MIP technology. Based on the above considerations, the complexity of MIPs and the perpetually increasingly publication rate render it as an interesting research domain for bibliometric analysis, in order to determine the underlying sources of influence and perform a methodological review of the attributes and the advances of this phenomenon.

#### Define the research objectives and research questions (step 2)

The goals of the current bibliometric study can be classified into two main pillars (Donthu et al. 2021) that are to (g1) *conduct a MIPs scientific literature performance analysis* and (g2) *apply a science mapping on MIPs within the 1990–2021 period*. At a first level (g1), we adopt an exploratory perspective focusing on evaluating performance indicators through an in-depth analysis of extracted metadata and the identification of the most active contributors at author, institution, country, and publication venue levels. At a second level (g2), the main focus is to provide a holistic roadmap related to the most impactful publications, analyze the social structure of the research constituents and how they interact to each other, and, finally, unveil topics of scientific activity that attracted the interest of the researchers throughout the examined period. To fulfill the above goals, we formulated the following research questions (RQs) (Table 1).

**Table 1:** Interpretation of the different types of bibliometric maps applied to answer research questions RQ1.1 to RQ2.3

RQ (s)	Purpose	Goals	Method	Metadata fields
[RQ_1.1_] What is the molecular imprinted polymers research landscape throughout the examined period and which are the temporal trends within the past decades?	RQ_1.1._ intends to present the outcomes of 30 years of scientific research on MIPs to allow for a deeper comprehension of the knowledge and structure pertaining to the various aspects relative to the research field and to observe scientific impact and potential temporal trends	(**g1**) Performance analysis	Descriptive statistics and exploratory data analytics	Timespan Sources Number of documents Number of documents per author Number of authors per document ratio
[RQ_1.2_] Which are the most active research contributors at author, institution, country, and publication venue levels?	RQ_1.2_ aims to explore the impact of the contributors to the field in the MIPs’ research community. To fully capture the scope of the contributors, we have divided them into different levels, with each level revealing different aspects of MIP research activity	(**g1**) Performance analysis	Descriptive statistics and exploratory data analytics	Timespan Sources Number of documents per author Number of authors per document ratio
[RQ_2.1_] Which are the most influential research publications?	RQ_2.1_ intends to determine which cited journals and authors have attracted the highest interest, considering that these publications and authors can be viewed as more influential in the related research community	*(****g2***) Science mapping	Mixed-type analysis/advanced multivariate data analytics/*Louvain* algorithm with *Salton’s normalization* (Aria and Cuccurullo 2017)	Average citations per documents/average citations per year
[RQ_2.2_] Which are the social interactions among the most prominent research contributors at author and country levels?	RQ_2.2_ aims to examine frequent collaborations between different contributors. Given that collaborations have often served as the basis for new research developments, they thus can be considered as the direct intellectual basis of the MIPs research field	*(****g2***) Science mapping	Mixed-type analysis/advanced multivariate data analytics/*Louvain* algorithm with *Salton’s normalization* (Aria and Cuccurullo 2017)	Co-authors per document ratio Collaboration Index
[RQ_2.3_] Which themes are the focal points of molecularly imprinting polymers research, and which trends can be extrapolated from them?	RQ_2.3_ aims to extract the semantic information hidden in the corpus of the examined studies in order to identify primary topics of interest synthesizing the body of knowledge related to MIPs research	*(****g2***) Science mapping	Exploratory analysis/*Latent Dirichlet Allocation* (LDA) (David M. Blei et al. 2003)	Author’s keywords (DE) Keywords plus (ID)

### Conducting phase

The *conducting phase* can be considered as a critical one, since it comprises the necessary actions and decisions dedicated to the identification and retrieval of the research activity focusing on the topic under investigation.

#### Identification of research (step 3)

This step is related to the establishment of predefined inclusion and exclusion criteria that should be met in order to identify the eligible set of candidate studies that will be used for further analysis. In the current study, we made use of an automated search strategy, since it is considered as the most effective one, when the scope is broad and the number of candidates is too large for manual search (Petersen et al. 2015). Regarding the selection of the digital libraries that will be used for performing the automated search strategy, we opted to utilize one of the most popular scientific databases, namely, the Scopus database, based on the following criteria: (*i*) the high coverage of research studies from a wide range of scientific domains and (*ii*) the availability of appropriate tools for performing systematic searches (Gusenbauer and Haddaway 2020; Kong et al. 2019). The final search string was consisted of the two following search strings that were “molecularly imprinted polymers” and “molecular imprinting.” Both search strings were applied on the titles, abstracts, and keywords of the articles, while the searching process was completed on April 2022.

#### Selection of studies (step 4)

After the application of the search string on the Scopus digital library, the identified initial set of candidate studies was further subjected to a filtering process based on specific *inclusion criteria* (IC) that should be met for qualifying a study to be included into the final set of the examined articles. The selection process was based on ICs taking into account metadata provided by Scopus. More specifically, the candidate study should be (*i*) solely of article document type excluding reviews, conference papers, book chapters, books, short surveys (*ii*) written in English, and (*iii*) published within the examined period (1990–2021). At this point, we decided to limit the period range and exclude all studies published during 2022, in order to mitigate potential threats stemming from the fact that the search process was conducted on April 2022.

#### Data extraction (step 5)

The next step involves the data collection process aiming at extracting all the necessary information related to each identified study that will be used, in turn, for data analysis purposes and synthesis of the body of knowledge. To this regard, a fully automated extraction strategy was adopted, exploiting the functionalities provided by Scopus in order to mitigate both subjectivity and bias threats.

Having in mind that the search process was conducted through the application of two independent search strings, the dataset comprised a total of 20,159 studies (10,581 and 9578 studies were returned from “molecularly imprinted polymers” and “molecular imprinting” search strings, respectively). Due to this fact, a de-duplication step based on the removal of studies presenting identical titles took place and resulted into the final set of 11,131 studies.

#### Data synthesis (step 6)

The final step of the *conduction* phase is associated to the selection and application of appropriate statistical and data analysis methodologies in order to fulfill the general goals of the study and provide straightforward answers to the posed RQs. Table 2 presents the evaluation of well-known performance indicators.

**Table 2:** Interpretation of the different types of indicators related to performance analysis

Type of metric	Measure entity characteristic	Interpretation
Number of documents	Productivity (Cucari et al. 2022)	Characterization of the research quantity (Song et al. 2021)
Average citations per documents/average citations per year	Scientific impact (Cucari et al. 2022)	The amount of citations and average citations which are frequently linked with the quality and impact of scholars (Song et al. 2021; Tang et al. 2018)
Author’s keywords (DE)	Identify the existing research themes (Zhang et al. 2016)	Relates to a set list of keywords that authors use to convey what their research was about (Tripathi et al. 2018)
Keywords plus (ID)	Represent the knowledge base embodied in the analyzed collection and explore the different thematics developed into the research domain (van Meter et al. 2004)	This metric refers to the total number of keywords created by Scopus, based on the titles, keywords, and abstracts of publications examined (Tripathi et al. 2018)
Number of documents per author	Productivity of authors (Arias-Ciro 2020)	The quantity of research works produced by the authors (Song et al. 2021)
Number of authors per document ratio	Authorship pattern (Aria et al. 2020)	This ratio assesses if researchers tend to produce single- or co-authored works, and it can be also used to interpret the average size of research teams (Aria et al. 2020)
Co-authors per document ratio	Information on direct and indirect connections of each author (E. Y. Li et al. 2013)	The frequency of an author’s appearances in a collection of documents (Aria et al. 2020)
Collaboration index	Trend towards multiple authorships in a discipline (Karpagam et al. 2011)	The nature and magnitude of a collaboration (Ajiferuke et al. 1988; Subramanyam 1983)

Regarding RQ_2.3_, the Latent Dirichlet Allocation (LDA) algorithm (David M. Blei et al. 2003) was performed on the corpus of both the titles and the abstracts of the collected studies in order to extract MIP research topics and thematic axes, which have attracted the interest of the research community, leveraging the semantic structure in the collection of documents and the interconnections between frequently co-occurring words. Regarding the deployment of LDA on the corpus of the collected studies, necessary pre-processing procedures (e.g., removal of stopwords, special characters and punctuation marks, transformation to lowercase, tokenization and stemming) were performed to ensure a LDA solution that would meet certain quality criteria by removing noise that is inherent in any collection of documents.

The primary goal of the LDA algorithm is the discovery of topics within a document collection or groups of words that are more frequent in a large number of documents. The number of topics is defined before the execution of the algorithm, and each word is assigned to each topic with a probability. Evidently, the highest the probability, the more likely it is that a word belongs to a specific topic in a higher degree than other topics. Hence, the LDA algorithm maps, in an automated manner, each word and each document of a corpus to the predefined number of topics, with higher or lower probabilities. The extracted topics, consisting of words from the corpus, can then be interpreted using expert judgment in order to detect motor research themes that drive MIP literature.

Concerning the decision about the number of extracted topics (denoted by $T$) which is a significant factor for the identification of interpretable and meaningful topics, a universally optimal value of $T$ cannot be defined in advance, as different values can be used depending on the research purposes and the size of the corpus, after careful experimentations and trials. In the context of our study, several experimental runs were conducted to determine the best value of $T$ through the evaluation of the derived LDA solutions via the *coherence score* metric showcasing that a value of $T=7$ extracted topics efficiently capture thematic patterns in the corpus of studies. Last but not least, *multi-dimensional scaling* was conducted on the extracted LDA solution aiming at the projection of the topics on a two-dimensional space that would, in turn, enable the visual exploration of both the distinctiveness of the topics and their semantic similarity.

The statistical analysis was mainly conducted using the programming language R (R a Language and Environment for Statistical Computing 2010) and two specific libraries, which are *bibliometrix* and *biblioshiny* packages (Aria and Cuccurullo 2017). Finally, the Gensim and PyLDAVis^1^ python packages were used for the fitting of the LDA models and the representation on a two-dimensional space, respectively.

## Results

In this section, we present the findings based on the posed RQs fulfilling the twofold goal of this bibliometric study.

### Performance analysis

- [RQ_1.1_] *What is the molecular imprinted polymers research landscape throughout the examined period and which are the temporal trends within the past decades?*

Table 3 presents the summary of the main information about the data. It is evident from Table 3 that the examined works were authored by 18,116 scholars, which corresponds to an average of 0.614 works per author. In fact, 1.48% of these works was written by a single author. Initial conclusions can be drawn by considering the authors and co-authors per document ratio and the collaboration index. Based on the authors per document ratio, it is possible to conclude that a work on MIPs was written by an average of 1.63 authors.

**Table 3:** Main performance indicators about the 1990–2021 collection

Main information about data	1990s	2000s	2010s	2020s	Whole period
Timespan	1990–1999	2000–2009	2010–2019	2020–2021	1990–2021
Sources	116	383	829	420	1146
Documents	317	1972	6995	1847	11,131
Average years from publication	24.9	16.2	6.91	1.5	8.17
Average citations per documents	97.1	56.03	29.64	7.035	32.48
Average citations per year per doc	3.703	3.238	3.827	2.65	3.524
References	5452	42,321	216,569	77,960	326,606
Document contents					
Keywords plus (ID)	1177	10,567	27,361	10,714	35,560
Author’s keywords (DE)	380	3138	10,562	4359	15,102
Authors					
Authors	516	3780	12,677	5054	18,116
Author appearances	1121	8144	36,030	10,212	55,507
Authors of single-authored documents	20	37	53	13	111
Authors of multi-authored documents	496	3743	12,624	5041	18.005
Authors’ collaboration					
Single-authored documents	31	53	68	13	165
Documents per author	0.614	0.522	0.552	0.365	0.614
Authors per document	1.63	1.92	1.81	2.74	1.63
Co-authors per documents	3.54	4.13	5.15	5.53	4.99
Collaboration index	1.73	1.95	1.82	2.75	1.64

During the whole period, the co-authors per document were estimated to approximately 4.99, while the number of authors per document was approximately 1.63. The discrepancy in the results can be attributed to the various ways authors are counted. If, for example, an author has published three different works, they will be included only once in the authors per document ratio, but they will be included three times in the co-authors per document ratio.

Due to the complicated nature of relationships between authors over a period of time, the exact nature of their collaborations is not easy to identify through these metrics. To this end, the collaboration index can be taken into account instead, which is defined as the total number of authors divided by the total number of multi-authored documents (Aria and Cuccurullo 2017). For the results appearing between 1990 and 2021, the collaboration index was estimated to be 1.64, thus confirming the findings from the other metrics. All the works had an annual average of 3524 citations.

**Fig. 2:**
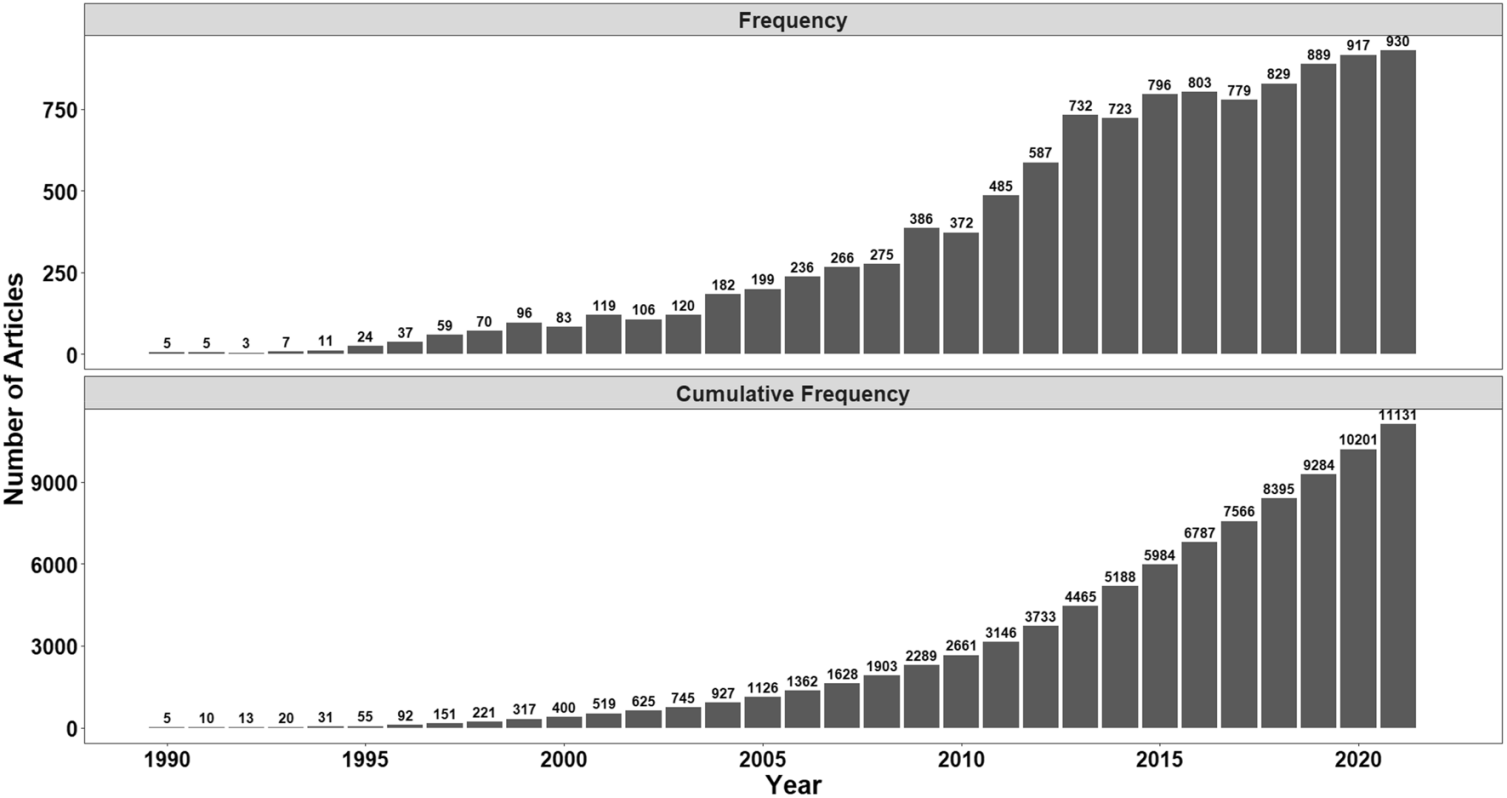
The number of publications from 1990 to 2021 in Scopus

The publication trends (the number of documents also included) reflect and measure the research activities and focus on a specific area. The increased production of the yearly outputs verifies the added focus on MIPs. The temporal distribution of publication output can confidently indicate the popularity, significance, and development trend of a research topic as depicted in Fig. 2, from which it can be observed that the cumulative number of publications is only 2289 during the first two decades (1990s, 2000s), but the rising trend apparently levels out and triples in number, exceeding 9000 by 2019.

The cumulative number of publications was 92 by 1996, while it reached 11,131 by 2021 and increased rapidly since 2000, whereas the maximum yearly number of publications reached 930. Additionally, the cumulative percentage of the number of publications showed that nearly 50% of cumulative publications were published after 2015, which means that during 2016–2021, the authors have contributed roughly half of all of the papers that have been published in MIPs during the examined period (1990 to 2021). The publication output displays an exponential rise which suggests that MIPs is a subject that is gaining a lot of attention in recent years. The increasing growth rate further suggests that the research on MIPs is still in the rapid development stage.

- [RQ_1.2_] *Which are the most active research contributors at author, institution, country, and publication venue levels?*

#### Author level

As shown in Table 4, Wang Y is the most productive author in MIPs with 328 publications followed by Zhang Y with 298. The productivity distribution of the authors was not uniform; only a limited number of authors produced the majority of the works.

**Table 4:** Leading authors in MIPs based on the number of publications

Ranking	Author	Articles	Articles fractionalized
1	Wang Y	328	56.40
2	Zhang Y	298	52.49
3	Li J	272	48.76
4	Li Y	270	47.09
5	Wang X	259	43.24
6	Wang J	251	46.56
7	Chen L	210	42.96
8	Wang S	205	37.01
9	Liu Y	199	34.00
10	Li X	195	39.22

**Fig. 3:**
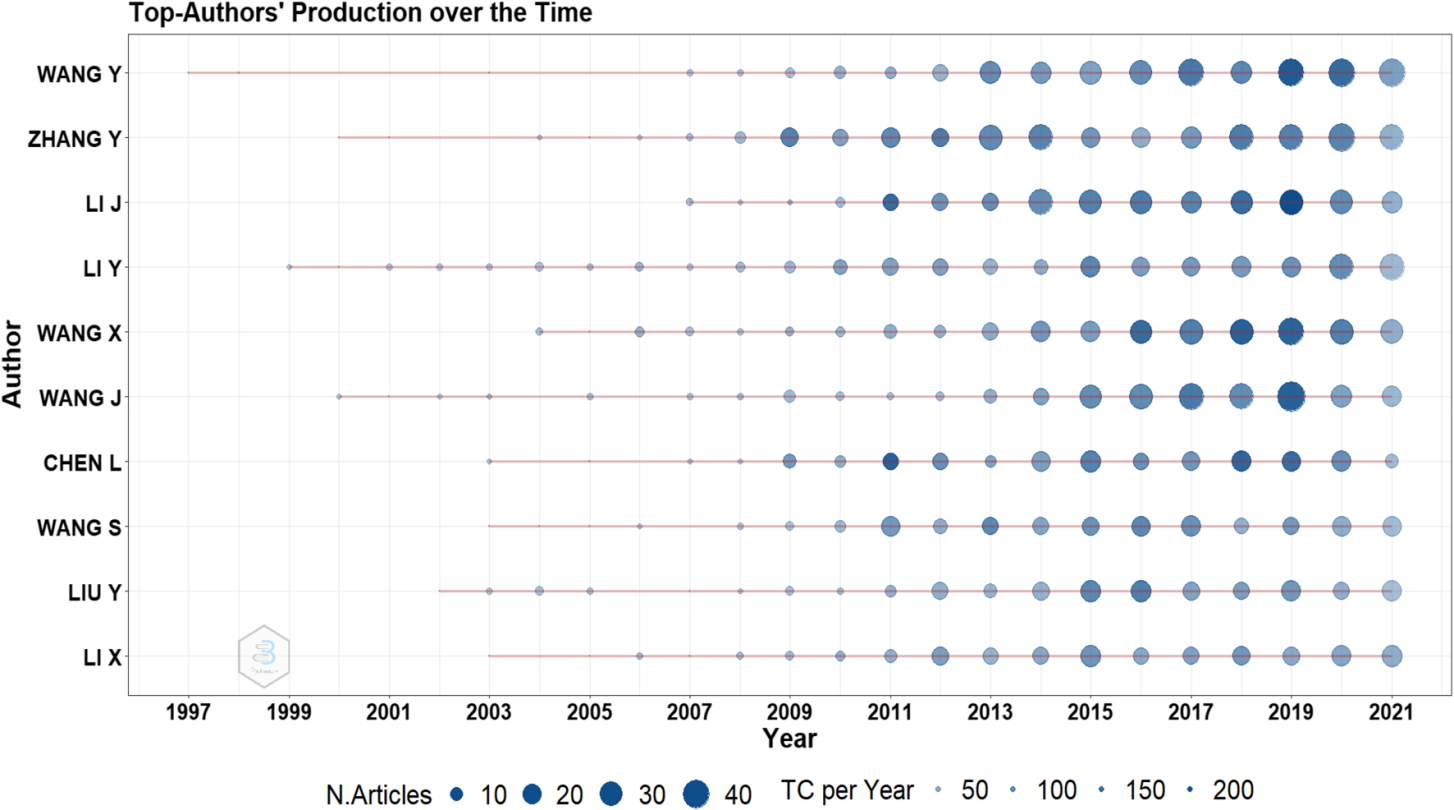
Author dominance over the time. Over the past 5 years, Wang Y has exceeded 40 publications annually with the exception for 2018, receiving a high number of citations

Figure 3 shows the most productive authors over time. The line depicts a researcher’s timeline; the bubble size corresponds to the author’s annual publications; the color intensity of the bubble is related to the total amount of annual citations (TC represents total citations in the figure legend); the first bubble on the line refers to the author’s first publication on the field; the larger the bubble, the more articles an author published yearly; deeper colors indicate higher citation frequency. From Fig. 3, we see that the most productive authors are Wang Y from 1997 to 2021, Zhang Y from 2000 to 2021, and Li J from 2008 to 2021.

#### Institution level

As shown in Table 5, Jiangsu University (China) has the highest number of publications on the topic (*n* = 391). In Europe, Lund University gets the highest number of total publications of 180 among the leading institutions of MIPs. With the exception of one research center, the remaining top 10 contributors are universities. Additionally, the majority of the leading institutions are from China.

**Table 5:** Leading institutions in MIPs

Ranking	Affiliation	Articles
1	Jiangsu University	391
2	Nankai University	248
3	Hacettepe University	241
4	Islamic Azad University	203
5	Lund University	180
6	Tianjin University of Science and Technology	118
7	Dalian Institute of Chemical Physics	111
8	Jilin University	109
9	Anadolu University	106
10	Cranfield University	103

#### Country level

**Fig. 4:**
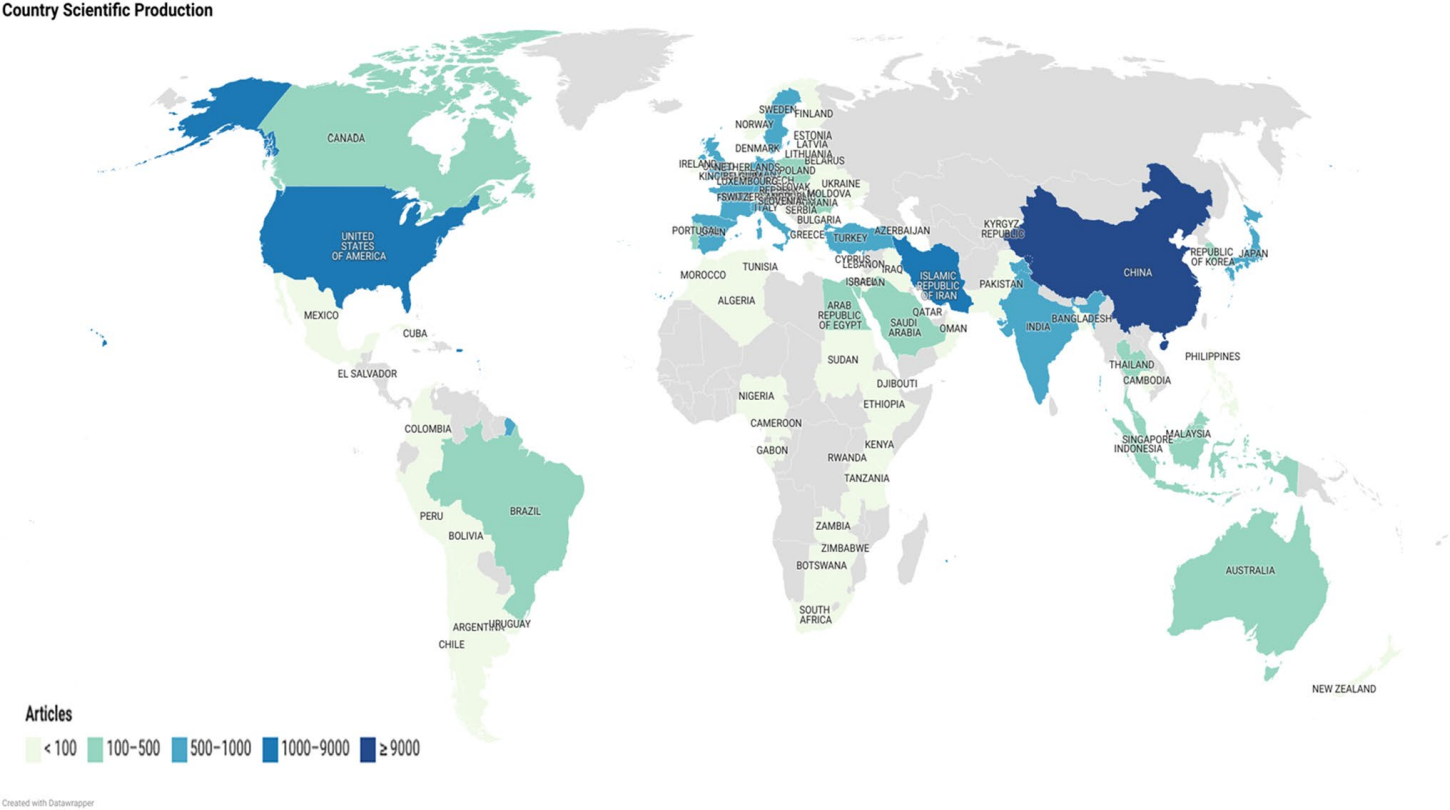
Geographical distribution of MIPs globally

The results of Fig. 4 and Table 6 show the geographical distribution of MIP research globally, the former depicting the scientific production per country, while the latter presenting information related to the corresponding authors’ countries. More specifically, and according to the respective research institute addresses, 88 countries were identified. The number of publications is represented by a gradient color in the map, i.e., the darker the color, the larger the publication number. In this regard, the number of publications for each country is estimated based on the sum of the country affiliations of all co-authors that contribute to a given publication.

**Table 6:** Corresponding author’s country

Ranking	Country	Articles	Freq	SCP	MCP	MCP_ratio
1	China	4420	0.422	4087	333	0.0753
2	Iran	728	0.070	683	45	0.0618
3	USA	622	0.059	499	123	0.1977
4	Japan	476	0.045	428	48	0.1008
5	India	351	0.034	325	26	0.0741
6	Spain	329	0.031	221	108	0.3283
7	Turkey	321	0.031	292	29	0.0903
8	United Kingdom	283	0.027	173	110	0.3887
9	Sweden	268	0.026	147	121	0.4515
10	Germany	261	0.025	166	95	0.3640

*SCP* single-country publications, *MCP* multiple-country publication, *MCP ratio* multiple-country publication ratio

According to the information gathered regarding the countries of the corresponding authors, these works primarily stem from China, which is the most important contributor with 4420 published works, followed by Iran (728) and the USA (622). These countries combined amount to more than 55% of the publications on MIPs. Within Europe, most of the work originates from the UK and Sweden with other countries such as Turkey, the UK, Germany, Italy, and France contributing moderately. In Asia, MIP research is mostly conducted in Japan, China, and Iran.

#### Publication venue

The studied 11,131 works were published in various academic journals. Table 7 lists the top 10 most active journals concerning the domain of MIPs. More specifically, the *Journal of Biosensors and Bioelectronics*, with 484 publications, is definitely an active venue, followed by the *Journal of Chromatography* (438 publications), and *Analytica Chimica Acta* (416 publications). *Biosensors and Bioelectronics*, possessing an impact factor of 10.62, is ranked as third out of 83 in *Chemistry Analytical* based on the fact that this journal has often published about topics related to MIPs. The *Journal of Chromatography* (IF = 4.75) publishes studies on sample preparation, and *Analytica Chimica Acta* (IF 6.55) aims at exploring, among other fields, sample treatment methods. The subject category “chemistry, analytical” appears 10 times in the top 10 most active journals. Each journal covered by Web of Science belongs to a minimum of one category, with each of those subjects covering a specific research topic. It should be noted that the Web of Science consists of roughly 250 distinct categories. Each article published in a journal will automatically be designated with the journal’s respective categories. The analysis results indicate that the published research outcomes on the MIPs obtain more attention in analytical chemistry and biochemical research methods than in pure chemistry journals.

**Table 7:** Top 10 most active journals on MIPs

Rank	Source	Subject category of the journal ^*^	#publications
1	*Biosensors and Bioelectronics*	Physics, electrochemistry, nanoscience & nanotechnology, biotechnology & applied microbiology, chemistry analytical	484
2	*Journal of Chromatography A*	Chemistry analytical, biochemical research methods	438
3	*Analytica Chimica Acta*	Chemistry analytical	416
4	*Sensors and Actuators B: Chemical*	Instruments & instrumentation –electrochemistry chemistry, analytical	397
5	*Talanta*	Chemistry, analytical	388
6	*Journal of Separation Science*	Chemistry, analytical	385
7	*Analytical and Bioanalytical Chemistry*	Chemistry analytical, biochemical research methods	252
8	*Analytical Chemistry*	Spectroscopy, chemistry, analytical	208
9	*Microchimica Acta*	Chemistry analytical	204
10	*Journal of Applied Polymer Science*	Polymer science	183

^*^Subject categories were retrieved from the 2021 Journal Citation Reports

- [RQ_2.1_] *Which are the most influential research publications?*

A high number of citations is usually indicative of the influence of the publication in a particular field; thus, the number of publications surpassing a set citation threshold is an excellent indicator of a publication’s level of influence (Merigó et al. 2015a, b). The top 10 publications are listed in Table 8.

**Table 8:** Top 10 most cited publications on MIPs (ranked by total number of citations)

Rank	Publication	Publication title/description	Total CT	TC per year	Normalized TC
1	Haupt K, 2000, *Chem Rev*	**Molecularly imprinted polymers and their use in biomimetic sensors**	1891	82.22	20.56
		This review determined that a primary trend in the biosensor sector relates to miniaturization and creation of multi-sensor arrays			
2	Vlatakis G, 1993, *Nature*	**Drug assay using antibody mimics made by molecular imprinting**	1606	53.53	4.61
		This work focused on the precise measurement of drug levels in human serum, with findings that can be compared to those procured through a prevalent immunoassay method			
3	Chen L, 2011, *Chem Soc Rev*	**Recent advances in molecular imprinting technology: current status, challenges and highlighted applications**	1321	110.08	30.37
		This critical review succinctly summarized the current status of molecular imprinting technologies, focusing on the notable progress of innovative imprinting methods, and the challenges faced, as well as the strategies and applications of MIPs			
4	Wulff G, 2002, *Chem Rev*	**Enzyme-like catalysis by molecularly imprinted polymers**	1309	62.33	15.26
		This review discussed the current status and the challenges in the preparation of enzyme-like catalysts through imprinting			
5	Norotte C, 2009, *Biomaterials*	**Scaffold-free vascular tissue engineering using bioprinting**	920	65.71	19.64
		The authors described the implementation of a rapid prototyping bioprinting for scaffold-free small diameter vascular reconstruction, which is not limited to tubular biological structures			
6	Ficetola GF, 2008, *Biol Lett*	**Species detection using environmental DNA from water samples**	824	54.93	16.79
		This work described the utilization of specified primers that can amplify short mitochondrial DNA sequences to track the presence of a frog (*Rana catesbeiana*) in controlled environments and natural wetlands			
7	Zhu G, 2012, *Nano Lett*	**Triboelectric-generator-driven pulse electrodeposition for micropatterning**	647	58.82	16.94
		This study described a novel robust generator that is easy to produce at low cost, but has an impressive electric output, and employs energy-harvesting technology to address pollutant degradation, corrosion protection, and water splitting			
8	Miyata T, 2002, *Adv Drug Deliv Rev*	**Biomolecule-sensitive hydrogels**	626	29.81	7.30
		This review highlighted all the significant prior art on the synthesis and applications of glucose-sensitive hydrogels that sustain swelling changes depending on glucose concentration			
9	Shi H, 1999, *Nature*	**Template-imprinted nanostructured surfaces for protein recognition**	623	25.96	6.61
		The present work proposed a method for imprinting surfaces with protein recognition sites, using radio-frequency glow-discharge plasma deposition to produce thin polymeric films surrounding proteins that have been coated with disaccharide molecules			
10	Yeo WH, 2013, *Adv Mater*	**Multifunctional epidermal electronics printed directly onto the skin**	574	57.4	16.26
		In this study, the use of advanced materials and integration techniques have been reported to achieve improved mechanics and strong bonds in epidermal electronic systems (EES) aiming to monitor body responses through and on the skin			

*Total TC* total citations, *TC per year* total citations per year, *normalized TC* normalized total citation

The requirement to distinguish publications based on the document types is important in scientometrics and research evaluations because each document type’s particular goals and contents are studied and cited differently, thus resulting in varied citation distributions. The distribution curve of each document type is different over time, and the same applies to the average citation speed and characteristics, depending on their communicative goal and knowledge gap to cover. For instance, articles are typically longer than letters, while reviews tend to have a larger number of references than an article (Donner 2017).

Porter et al. (Porter et al. 2019) suggested that new research areas could be cited more often; however, Thelwall et al. (Thelwall and Sud 2021) argued that the impact of novelty in citations is significantly different between fields. Waltman (Waltman 2016) confirms this fact and proposes that one of the main principles of citation analysis is that citation statistics of published works from non-relevant fields should not be subject to direct comparisons, due to the sizable differences between research fields in terms of citation density, i.e., the average citations per publication. In the same vein, it is frequently suggested that citation statistics of different document types should not be compared, because some of them, for example, review articles, have the tendency to attract significantly more citations than typical research articles (Waltman 2016).

In the case of our study, for instance, two out of the top three most cited publications are review articles by Haupt et al. (Haupt and Mosbach 2000) and Chen et al. (Chen et al. 2011).The present study examined the declared document types provided by Scopus and discovered some slight divergences, where review articles were classified to the research “article” category and were thus identified differently compared to how they were by the official journal websites, which could potentially affect the accuracy of the bibliometric analysis (Franceschini et al. 2016). Chiu and Ho (Chiu and Ho 2007) proposed that the number of citations of a publication by others is not indicative of the publication’s quality but that it highlights its visibility instead. Furthermore, it is commonly accepted that open access journal publication citations are incrementally growing (Whipple et al. 2013). A rather small number of publications with a comparatively high total number of citations statistically verify that works in the review paper category have higher chances of receiving more citations. Evidently, the amount of citations is strongly correlated to the elapsed time since its original publication (Qiu and Chen 2009). Obviously, older works have higher chances to be more actively cited than newer ones, but this does not inhibit the most recent publications from having a significance influence on their field (Milfont and Page 2013).

If the content of the ten most frequently cited publications is examined, it can be seen that they cover a variety of topics which implies a wide range of research themes and the multidisciplinary nature of MIP research. However, by reviewing the top 10 most frequently cited publications, it becomes evident that 70% belong to the chemistry multidisciplinary field.

- [RQ_2.2_] *Which are the social interactions among the most prominent research contributors at author and country levels?*

**Fig. 5:**
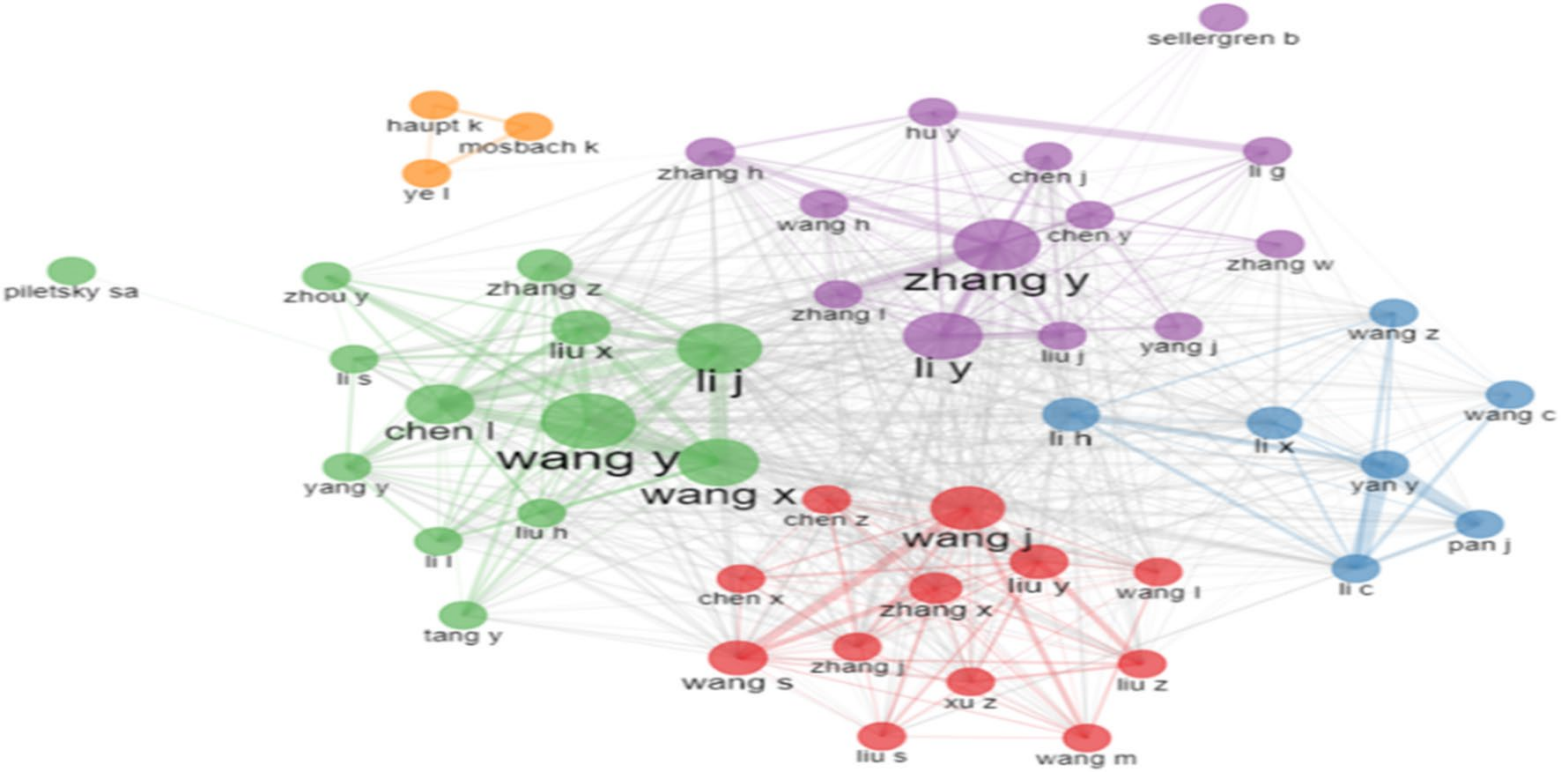
Cooperation network between different authors on MIPs

In Fig. 5, the collaboration network of the authors is presented, in which nodes (circles) represent authors and their size depends on the amount of their published works, whereas curves represent the connections between different authors. The colors in the visualization networks designate cooperation clusters.

Overall, the nodes of the collaboration network seem to follow a pattern, with one central node (author) that is active and has multiple publications in the field (Wang Y in the green cluster, Zhang Y in the purple cluster, Wang J in the red cluster) and other nodes mainly collaborating with this node and among each other. In addition, a triad of isolated nodes is observed (yellow cluster) which may indicate researchers working on niche subjects or an efficient group that frequently collaborates without external interventions.

One important aspect that demands more scientific attention is the observation that the most active researchers in MIP research may not necessarily be the most influential ones, as confirmed by Figs. 2 and 5. The literature on MIP research has been relatively recent, with a significant impact observed especially after 2016. This increase in impact could be attributed to the growing activity within the MIP research community during that period, as shown in Fig. 2. However, despite this growing activity, there seems to be a stronger knowledge communication among active authors than a knowledge flow from highly influential authors to active authors worldwide (Fig. 5).

It is noteworthy that even highly impactful articles, as observed by Ponomarev et al., might experience a decline in citations over time due to the memory of aging effects. (Ponomarev et al. 2012). Nonetheless, earlier published process MIP research, such as the work of Haupt et al., has continued to receive significant scholarly attention and continues to inspire new ideas. This discovery suggests that research scholars frequently refer to impactful MIP literature from the period before the MIP community experienced significant growth, instead of solely focusing on recent works from high-profile authors (Yang and Wang 2015).

However, it is essential to bear in mind the lifecycle of research articles, where citations accumulate over time (Ponomarev et al. 2012). Relying solely on the number of citations in bibliometrics can lead to an underappreciation of emerging research topics or authors, especially for more recently published articles that have not yet had sufficient time to accumulate a high number of citations. This consideration is crucial to avoid potential biases in the analyses, as research published a longer time ago may receive higher citation counts simply due to its age.

Among the most active authors, *Biosensors and Bioelectronics* journal is the most prominently represented, with 5 publications. Following closely are *Chromatography A* and *Talanta* with 4 publications each. The journal *Analytical Chemistry* also makes an appearance with 3 publications.

**Fig. 6:**
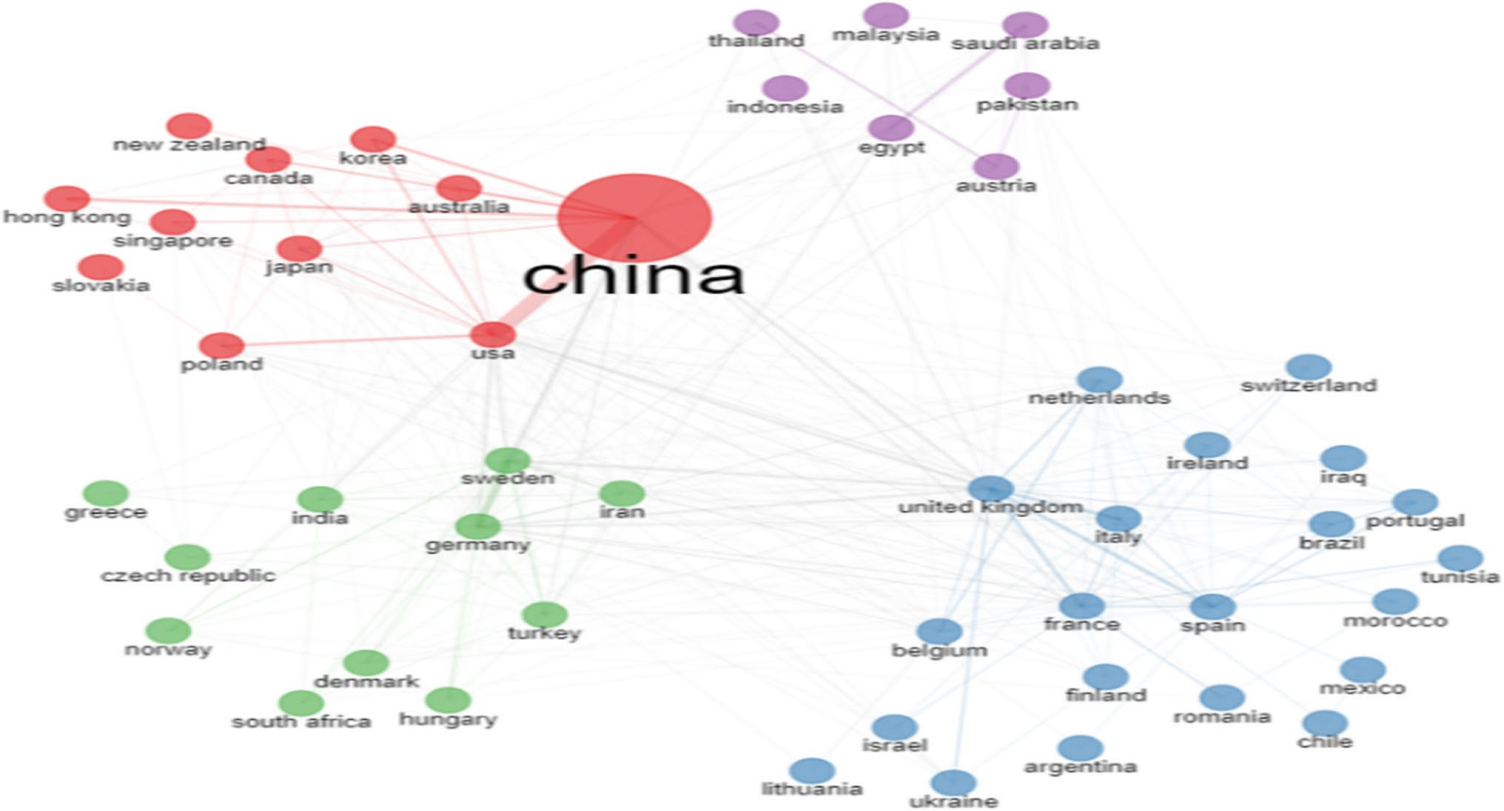
Country collaboration network in MIPs’ publications

The country/region collaboration network in the studied research field was also produced and analyzed (Fig. 6). Each node’s color represents a different collaboration cluster. The node’s diameter indicates the related publication number, and the density of the links depicts the level of international collaborations; i.e., the larger the node is, the more productive the country/region is, and the thicker the link connection is, the more intense the cooperative relationship between the linked nodes.

A first interesting remark derived from the exploration of Fig. 6 indicates that China has been at the forefront and plays the predominant roles in MIPs. The cluster gathering around China has four other major contributors: the USA, Hong Kong, Poland, Canada, and Korea. Similarly, the close collaboration countries/regions with the UK are Netherlands, France, Spain, etc. Additionally, among the close collaboration countries/regions, China and the USA have the most immediate cooperation and research relationship in MIPs. Generally, future international collaborations should be further cultivated to disseminate knowledge worldwide. As reported in other scientific research fields, collaborating countries are often geographically correlated, and cluster around the most productive representatives in terms of publication output (Zheng et al. 2016).

- [RQ_2.3_] *Which themes are the focal points of molecularly imprinting polymers research, and which trends can be extrapolated from them?*

In order to extract prominent topics of research, we further leveraged the textual content found in the titles and abstracts of the collected studies. To this regard, the results of the LDA model are presented in Table 9. Each topic is associated with a set of relevant terms (excluding common terms used for the identification of the collection of the studies, e.g., “mip,” “molecular,” “imprinted”) that was the basis for extracting, to the best our ability, a short description reflecting the thematic axes of the research landscape. Moreover, we computed the share metric that provides straightforward insights concerning topics that have gained the interest of the research community to a greater or lesser extent. This specific measure takes into consideration the whole distribution of the membership values for the set of relevant terms indicating the proportion of studies related to a specific topic (Barua et al. 2014).

**Table 9:** Results of the LDA model

Topic interpretation	Relevant terms	Share%
**Topic 1:** MIP techniques	Extract, recovery, determination, solid-phase extraction, water, detection, liquid, analyte, limit, sorbent	36.02
**Topic 2:** MIPs with catalytic activity	Hydrogel, catalyst, lens, ocular, hydrolysis, GSH, imidazole, zeolite, HA, lactose	4.14
**Topic 3:** MIP photocatalysts	Magnet, membrane, Fourier-transformed-infrared, microscope, x-ray, electron, spectroscopy, transmission, diffraction, scan	12.59
**Topic 4:** MIP sensors	Sensor, detection, electrochemical, electrode, sensitivity, fluorescence, carbon, base, limit, film,	38.62
**Topic 5:** MIP in biomedical applications	Protein, target, specificity, nanoparticle, strategy, drug, substrate, active, material, biomarker	57.68
**Topic 6:** Synthesis and performance of MIPs for environmental applications	Absorption, selectivity, acid, monomer, preparation, capacity, template, polymer, synthesis, bind	72.71
**Topic 7:** MIPs in biological applications	Cell, cancer, tumor, expression, immunity, patient, infection, tissue, sialic, gene, breast	5.52

The examination of the share metric indicates that topic 6 (synthesis and performance of MIPs for environmental applications) can be considered as the most dominant topic across the set of the studies with a share value of 72.71% whereas topic 5 (MIP in biomedical applications) (share (topic 5) = 57.68%).

**Fig. 7:**
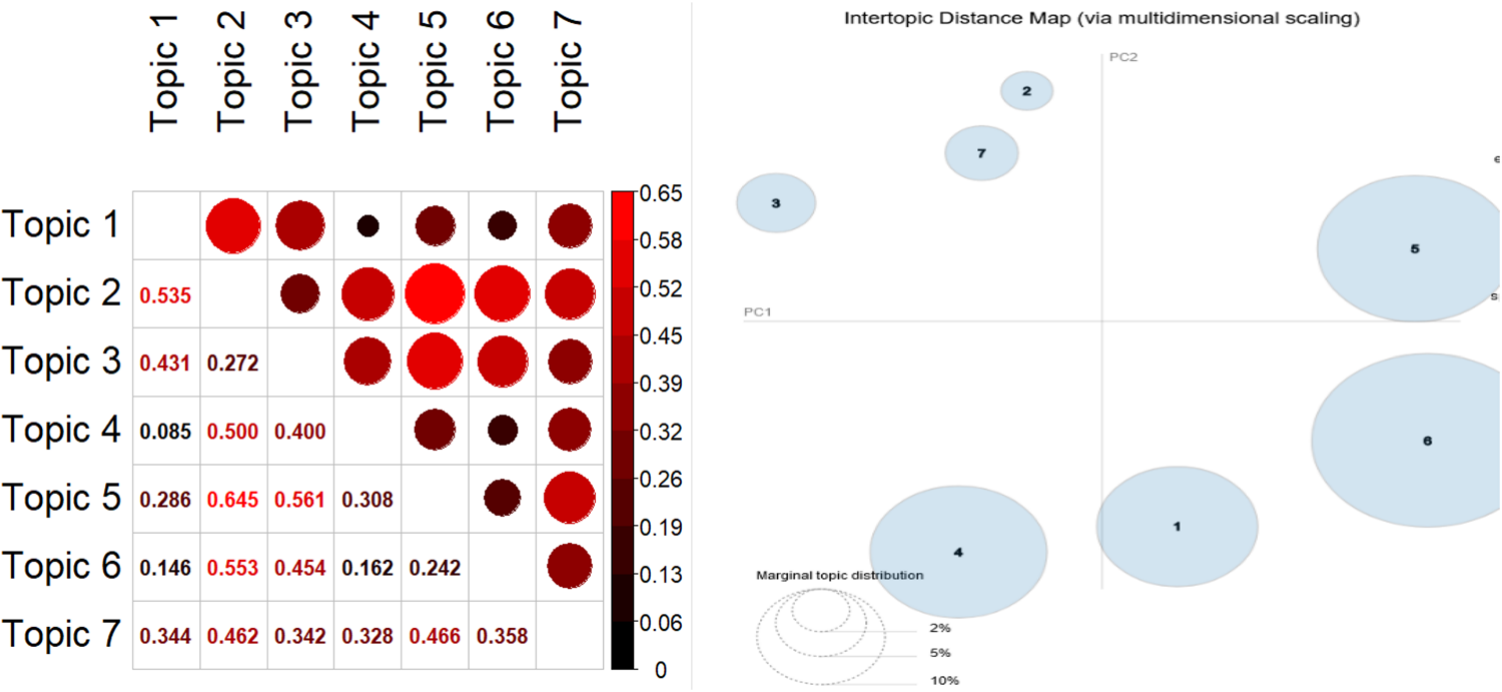
Topic modeling analysis

Regarding the quality of the LDA solution, apart from the coherence metric score used for the selection of the optimal number of topics, we also made use of the multi-dimensional scaling approach, a well-known data reduction technique, projecting the extracted topics on a two-dimensional space. The graphical representation of the topics can be used, in turn, for assessing the quality of the LDA model, because a good LDA solution should be visualized by large-sized and non-overlapping circles that are placed into the four quadrants of the plot. In the right panel of Fig. 7, we presented the graphical representation of the topics after the conduction of the multi-dimensional scaling method indicating coherent and efficiently distinguishable topics.

In addition, the magnitude of each circle size provides a glimpse of the prevalence of the corresponding topic in the collection of the examined studies facilitating the identification of prominent thematic axes. Finally, the careful inspection of the relative position of topics represented by different circles and their distances can be used for assessing the semantic association between the extracted topics. The pair-wise dissimilarities were also evaluated via the Jensen–Shannon divergence, a measure that quantifies the distance between two probability distributions, representing the distributions of two extracted topics on the corpus of MIP publications in our case.

The results are presented in the left panel of Fig. 7, from which we can observe that topic 1 (MIP techniques) and topic 4 (MIP sensors) are highly associated themes presenting the smallest Jensen–Shannon divergence value, a fact that is also graphically displayed through the contingency of the corresponding circles into the two-dimensional plot. Additionally, topic 6 (synthesis and performance of MIPs for environmental applications) is closely related to both topic 1 (MIP techniques) and topic 4 (MIP sensors), whereas the remaining topics are positioned far away from the rest, a fact that may indicate a small degree of semantic interrelation among these topics. This finding validates the fact that MIP research is indeed a multidisciplinary field that has attracted the attention of multiple scholars from various backgrounds, and the developed publications deal with a plethora of different subjects that may, or may not, be correlated with each other.

The interrelation between topic 1 and topic 6 and its projection on the two-dimensional plot (Fig. 7), indeed, confirm the strong semantic association between topic 1 and topic 6. In this regard, when two LDA topics seem to be close in terms and subject, a usual mitigation step is to merge them into a broader topic for interpretation purposes. Thus, while the extracted topics of an LDA solution offer valuable information via data-driven methods, delving into the examined subjects of each topic through more in-depth and detailed scoping could offer greater insights. Upon manual examination of representative studies from each topic, we observed a varying degree of distinctiveness among them, with some showing considerable overlap. For instance, one topic is centered around MIP techniques, while another topic focuses on environmental applications.

In this regard, we decided to further, manually, categorize topics into more generic themes based on the results derived from the screening of the titles and abstracts of the representative studies that belong to each topic by initially identifying the most common MIP-related subject of discussions. If a topic was more relevant to a specific subject, it was treated as a standalone topic, while if multiple topics shared a common subject at an abstract level, they were assigned to a broader theme accordingly. As an example, topic 1 is more relevant to “MIP techniques,” while topic 6 mostly refers to “synthesis and performance of MIPs.” They could however be merged in a broader theme under the umbrella of “environmental applications.”

### Environmental application theme

#### Topic 1: MIPs’ techniques

In the first topic, the main terms revolved around MIP techniques, with the most frequent words being “extract, recovery, determination, solid-phase extraction, water, detection, liquid, analyte, limit, and sorbent.” This word composition strongly suggests an environmental emphasis in the articles, closely tied to MIP techniques. Several studies can be pointed out in this topic. Tao Zhao et al. successfully employed a combination of solid-phase extraction with capillary electrophoresis (SPE-CE) to determine pesticide residues in vegetables. The study specifically focused on quantitative detection of trichlorfon residues in leek samples. The results demonstrated the efficacy of this methodology, as the findings matched those obtained through gas chromatography (Zhao et al. 2014). Xusheng Ge conducted a study in which they devised a sample preparation procedure employing an accelerated solvent extraction (ASE) method, followed by a cleaning process using melamine molecularly imprinted polymers solid-phase extraction (MISPE). Their innovative approach resulted in a highly sensitive ASE-MISPE ultra-performance liquid chromatography (UPLC) method, allowing for effective separation and simultaneous determination of dicyandiamide (DCD), cyromazine (CYR), and melamine (MEL) in complex animal tissue foods (Ge et al. 2016).

#### Topic 6: Synthesis and performance of MIPs for environmental applications

The sixth topic contained terms such as “absorption, selectivity, acid, monomer, preparation, capacity, template, polymer, synthesis, and bind.” Topic 6 seems to entail publications on performance of MIPS for environmental applications. Terms such as “capacity” and “selectivity” strengthen the latter assumption. Based on our findings, this topic appears to be the most expanding among all identified topics. Furthermore, it is evident that this topic has witnessed substantial growth. We believe that this may be due to an intense increase in environmental pollution, which is also reflected in scientific initiatives. The following two studies can be included among the most representative studies. Muhammad Ali Zulfikar et al. in 2018 focused on microwave-assisted organic synthesis method for the adsorption of methylene blue (MB), a cationic dye from aqueous solutions. The results confirmed that the dye MIP could be used for the removal of cationic dyes from wastewater (Zulfikar et al. 2018). Three years later, Zhiyong Zhou et al. successfully synthesized a new rubidium ion-imprinted polymer using bulk polymerization. The produced material exhibited remarkable sorption capacity and specific recognition ability, enabling the efficient separation of rubidium ions from salt lake brines (Zhou et al. 2021).

### Biomedical application theme

#### Topic 4: MIP sensors

The fourth topic was dominated by the most commonly used terms such as “sensor, detection, electrochemical, electrode, sensitivity, fluorescence, carbon, base, limit, and film.” The term “sensor” prevailed, while the others provided additional information. Increase in articles centered around sensors can be attributed to the growing scientific interest in biomedical topics. One of the relevant studies by Hui Jin developed a MIPs/rGO-AgNPs/NF sensing system as a novel MIP electrochemical sensor of gastrodin. The novel and straightforward MIP electrochemical sensor, MIPs/rGO-AgNPs/NF, demonstrated effective detection of GAS in real biological samples, showcasing excellent detection stability and recovery during the process (Jin et al. 2018). In 2017, Zhao Wang devised an innovative sensor by creating an antimony-doped tin oxide-silica composite sol on a glassy carbon electrode, which was further modified with single-walled carbon nanotubes. This sensor was effectively used for detecting norepinephrine concentration in human blood serum samples. The study revealed good reproducibility, along with high stability and recovery of the sensor (Wang et al. 2017).

#### Topic 5: MIP in biomedical applications

Topic 5 consisted of terms such as “protein, target, specificity, nanoparticle, strategy, drug, substrate, active, material, and biomarker.” The composition of these terms suggests a close relationship to biomedical applications. The conventional enzyme activity assay is affected by evident interference from the sample matrix. Xiaodong Bi et al. introduced an innovative enzyme activity assay format designed to eliminate the influence of the sample matrix effectively. The key element of this approach involves a 96-well microplate that has been modified with a MIP prepared using a new approach called boronate affinity-based oriented surface imprinting. The boronate affinity MIP effectively preserves the enzymatic activity of glycoprotein enzymes, allowing the enzyme captured by the MIP to be directly utilized for the activity assay (Bi and Liu 2014). Zijun Bie et al. presented an innovative technique called “precision imprinting with alternative templates” for the synthesis of glycan-specific MIPs. The approach involves the use of glycopeptides with the desired peptide length immobilized on a boronate affinity substrate as alternative templates. Precision imprinting is then performed to create a thin layer covering the glycans to the desired thickness. This approach was proven to be highly versatile and effective. It is particularly significant for recognizing O-glycans since enzymes capable of releasing O-glycans from O-linked glycoproteins are limited. (Bie et al. 2018).

#### Topic 7: MIPs in biological applications

We have labeled the seventh topic as *MIPs in biological applications*. Additionally, other terms have been identified as complementary to this topic, including “cell, tumor, expression, immune, patient, infection, tissue, sialic, gene, and breast.” While this topic represents a relatively small portion of the overall content, its significance has increased over time. Back in 1990, this topic was practically negligible, but it has gradually grown since then. Megha Patel focused at monitoring the effects of sialic acid-molecularly imprinted polymers (SA-MIPs) on morphology and motility of the epithelial type MCF-7 and the highly metastatic MDAMB231 breast cancer cell lines, using digital holographic cytometry (DHC). SA-MIPs have a significant impact on the motility, morphology, and viability of both MCF-7 and MDAMB231 cell lines. Dynamic holographic cytometry (DHC) proves to be a potent tool for analyzing the motility and morphology differences between cell lines and for understanding the cellular responses induced by SA-MIPs treatment (Patel et al. 2020). Clinical tumors frequently exhibit high levels of sialic acid (SA) expression in tumor cells, which is closely linked to an elevated invasive potential and often corresponds to a poorer prognosis (El-Schich et al. 2021).

### Catalysis group

#### Topic 2: MIPs with catalytic activity

The second topic comprised terms such as “hydrogel, catalyst, lens, ocular, hydrolysis, imidazole, zeolite, and lactose.” The nature of these terms indicates a close association with catalysts. In comparison to other topics, this topic has a lower share value, likely because molecularly imprinted catalysts are still in the proof-of-concept stage (Muratsugu et al. 2020). In 1994, Katsutoshi Ohkubo et al. presented the initial instance of an imidazole-containing polymer that was molecularly imprinted with a transition state analog, demonstrating efficient homogeneous esterolytic catalysis. Surprisingly, the catalytic activity of this TSA-imprinted polymer in the Michaelis–Menten pathway was lower than expected, which was attributed to an unfavorable ΔS# factor. This was likely due to the polymer catalyst’s cavity not efficiently facilitating the lowering of the transition state for the hydrolysis of the short-chain substrate (Ohkubo et al. 1994).

#### Topic 3: Molecularly imprinted photocatalysts

The third topic primarily revolved around terms such as “magnet, membrane, Fourier-transform infrared, microscope, x-ray, and electron. Given its diverse meaning, we opted for the broader name “photocatalysts” for this topic. Despite its importance, this topic is relatively small-sized and has a smaller share compared to others. Yang Liu successfully synthesized molecularly imprinted polymer-coated Co-doped TiO2 nanocomposites (MIP/Co-TiO_2_ nanocomposites) using a surface molecular imprinting technique. Rhodamine B (RhB) was used as the template molecule, and p-phenylenediamine served as the functional monomer. The MIP/Co-TiO_2_ nanocomposites demonstrated a higher photo-degradation rate for RhB compared to the non-imprinted Co-doped TiO_2_ nanocomposites (NIP/Co-TiO_2_ nanocomposites). Furthermore, the MIP/Co-TiO_2_ nanocomposites exhibited excellent stability and reusability under irradiation (Y. Liu et al. 2016).

This study is liable to certain limitations, which will be presented using the classification schema proposed by Ampatzoglou et al. (Ampatzoglou et al. 2019) that identifies three main categories in secondary studies that act as limitations to the (*i*) *study selection*, (*ii*) *data*, and (*iii*) *research* validity. The study selection validity category encompasses limitations invoked in the search process and filtering phases. Regarding the strategy for the identification of the candidate studies, we made use of an automated search process using a well-known digital library to eliminate possible research bias that is inherent during a manual search process. As far as the selection of the digital library is concerned, and taking into consideration the inter-disciplinary nature of the current study, we opted to utilize Scopus, due to its broad coverage of research activity over a wide range of subject areas. Another limitation related to the identification of relevant studies concerns the construction of the string used in the search process, and for this reason, we decided to use two independent broad search strings. Moreover, we decided to apply the above search strings using separate time slices, so as to overcome the restriction of the Scopus digital library allowing the exportation of metadata fields for a limited number of studies. To that end, a data de-duplication process was conducted in order to identify and exclude identical studies retrieved by the application of the independent search strings described above.

Concerning the data validity limitations, we based the inferential process on metadata fields provided by Scopus to eliminate possible bias and conflicts stemming from the manual inspection of studies. Additionally, a subset of metadata was, finally, used for analysis purposes fulfilling the goals and objectives of the current study. Regarding the limitations to the research validity, the methodology that was followed is fully described along with the decisions made in subsequent steps (third section), enabling the reproducibility and replicability of the study. Moreover, the analysis was based on a large sample of collected studies, and thus, any limitation related to the generalization of the extracted findings is mitigated to a large degree. Finally, the data analytics approaches used for providing answers to the posed research questions are fully implemented in well-known open sources languages (i.e., R and Python) and well-studied libraries (bibliometrix, biblioshiny, PyLDAVis) mitigating potential problems resulted from the manual evaluation of performance indicators and/or user-defined code for science mapping purposes.

## Conclusions and perspectives

MIPs offer significant advantages over alternative adsorbent materials. By combining the appropriate monomer, crosslinker, and porogen, MIPs can be designed to be highly flexible and adaptable to various target molecules.

Apart from MIPs, a similar concept is that of ion-imprinted polymers (IIP) which was first introduced by Nishide and Tsuchida in 1976 (Nishide and Tsuchida 1976). These polymers differ from MIP as they utilize metal ions as substitutes for template molecules (Fu et al. 2015). Ion-imprinted polymers belong to the class of MIPs and exhibit exceptional selectivity and adsorption capacity towards cationic or anionic analytes (Mamo et al. 2020). While both IIPs and MIPs share the advantageous features of molecular imprinting technology, such as structure predictability, recognition specificity, and application universality, IIPs specifically target ions rather than molecules in the imprinting process (Alshuiael and Al-Ghouti 2022; Fu et al. 2015). IIPs are synthesized using a molecular imprinting synthetic approach where a template ion or molecular ion interacts with functional monomers through electrostatic and coordination interactions. The recognition sites in IIPs demonstrate higher affinity and selectivity towards template ions due to factors such as coordination geometry, ligand types, coordination numbers, cavity size, and ion radius and charge (Mamo et al. 2020).

In MIPs, the interaction between the template molecule and functional monomers relies on hydrogen bonds or van der Waals interactions, whereas in IIPs, the interaction between the metal ion and functional ligands or monomers occurs through coordination bonds, as they form metal complexes. Therefore, the careful selection of suitable ligands or monomers is crucial for complex formation (Adauto et al. 2020). One of the advantages of IIPs over most MIPs is their compatibility with aqueous media due to special coordination or electrostatic interactions. IIPs are particularly effective in identifying water-soluble ions, including heavy metals and radioactive elements that raise increasing concerns (Fu et al. 2015).

Over the years, there has been a noticeable increase in the number of published papers related to MIP research. This trend can be attributed to several factors. One significant reason for this surge in publications is the growing interest of researchers in the field (Ellegaard and Wallin 2015).The studies published in MIPs grew more dominant through this period probably due to the growth encouraged by a better awareness of the promising applications of molecular imprinting based technologies (Nicholls et al. 2001) and because the essence of the pre-arrangement in non-covalent imprinting (with various chemical capabilities and a comparatively simpler synthesis process) became a milestone that unlocked an array of applications in multiple scientific disciplines (Herrera-Chacón et al. 2021). Another reason is that the considerable increase in the number of researchers has resulted in a surge of publications and intensified competition in academic circles (Hou et al. 2022). The growing number of academic journals (Fire and Guestrin 2019) has further contributed to a “publish or perish” mentality among researchers, giving rise to the dilemma of “quality or quantity” in their studies. Furthermore, the expansion of scientific journals has been exponential, increasing from 10 at the end of the seventeenth century to a staggering 100,000 at the end of the tewentieth century (Ghasemi et al. 2022). The emergence of “predatory publishers” since 2010 — those without standard editorial boards, lacking proper peer review, and charging exorbitant publication fees — has exacerbated the situation (Grudniewicz et al. 2019; Kanna 2018). The number of papers published in predatory journals has grown dramatically over the years, with approximately 53,000 in 2010, 420,000 estimated in 2014, and around 10,000 predatory journals listed on Beall’s list by the end of 2016, approaching the number of journals in the *Journal Citation Reports and Directory of Open Access Journals* (Grzybowski et al. 2017).

The evaluation further revealed who stand on the frontier on the area of MIPs:

- Wang Y, Zhang Y, and Li J. are the most productive authors. They are central nodes in the author cooperation network; i.e., other authors are connected to them directly or indirectly. The authors are affiliated to China and publish in the research area of MIPs.
- Among the most active authors, *Biosensors and Bioelectronics* journal is the most prominently represented, with 5 publications. Following closely are *Chromatography A* and *Talanta* with 4 publications each. The journal *Analytical Chemistry* also makes an appearance with 3 publications.
- The *Journal of Biosensors and Bioelectronics*, with 484 publications, is definitely an active venue, followed by the *Journal of Chromatography* (438 publications) and *Analytica Chimica Acta* (416 publications).
- China, Iran, and the USA are the most productive countries in terms of volume of publications. In the cooperation network, other countries are connected (directly or indirectly) to these leading countries.

Some positive aspects could be concluded according to the results of the bibliometric analysis. Firstly, it can be deducted that there is abundant collaborative research in the field of MIPs. Collaboration, and especially the indicator of co-authors per document, grew over the past 3 decades, while, recently, papers with a single author have declined substantially. Indeed, the authorship pattern study suggests that multi-authored articles overshadowed single-author publications, a phenomenon which became more apparent over the past few years; however, the increase of multi-authored articles is limited to articles with two or three authors.

The trends, patterns, developments, gaps, and research directions identified through the presented analysis can serve as a valuable foundation for such reviews. It is hoped that researchers and international scholars can utilize the insights obtained from this analysis to identify potential areas for future collaboration. This collaborative effort has the potential to invigorate the research domain, introduce new ideas, concepts, and methods and ultimately enhance productivity, improve the quality and acceptance of solutions for various complex challenges in the field, and further enhance the performance of MIPs. However, there are still some clear gaps in our knowledge, which can be addressed from the disciplines of science and engineering. The growth rate implies that this behavior will carry over in the near future, which makes this an appealing topic for both current and future research. The findings of this work verify that MIPs are still a growing area of research, in which there are essentially no consolidated research patterns.

## Data Availability

Not applicable.

## Footnotes

1 https://github.com/bmabey/pyLDAvis (Accessed Nov. 09, 2022).
